# MR-detectable metabolic biomarkers of response to mutant IDH inhibition in low-grade glioma

**DOI:** 10.7150/thno.47317

**Published:** 2020-07-09

**Authors:** Abigail R Molloy, Chloé Najac, Pavithra Viswanath, Aliya Lakhani, Elavarasan Subramani, Georgios Batsios, Marina Radoul, Anne Marie Gillespie, Russell O Pieper, Sabrina M Ronen

**Affiliations:** 1 Department of Radiology and Biomedical Imaging, University of California San Francisco, San Francisco, CA, USA; 2 Brain Tumor Center, University of California San Francisco, San Francisco, CA, USA; 3 Department of Neurological Surgery, Helen Diller Research Center, University of California San Francisco, San Francisco, CA, USA

**Keywords:** IDH1 mutation, low grade glioma, hyperpolarized ^13^C magnetic resonance spectroscopy, AG-881, AG-120

## Abstract

Mutations in isocitrate dehydrogenase 1 (IDH1mut) are reported in 70-90% of low-grade gliomas and secondary glioblastomas. IDH1mut catalyzes the reduction of α-ketoglutarate (α-KG) to 2-hydroxyglutarate (2-HG), an oncometabolite which drives tumorigenesis. Inhibition of IDH1mut is therefore an emerging therapeutic approach, and inhibitors such as AG-120 and AG-881 have shown promising results in phase 1 and 2 clinical studies. However, detection of response to these therapies prior to changes in tumor growth can be challenging. The goal of this study was to identify non-invasive clinically translatable metabolic imaging biomarkers of IDH1mut inhibition that can serve to assess response.

**Methods:** IDH1mut inhibition was confirmed using an enzyme assay and ^1^H- and ^13^C- magnetic resonance spectroscopy (MRS) were used to investigate the metabolic effects of AG-120 and AG-881 on two genetically engineered IDH1mut-expressing cell lines, NHAIDH1mut and U87IDH1mut.

**Results:** ^1^H-MRS indicated a significant decrease in steady-state 2-HG following treatment, as expected. This was accompanied by a significant ^1^H-MRS-detectable increase in glutamate. However, other metabolites previously linked to 2-HG were not altered. ^13^C-MRS also showed that the steady-state changes in glutamate were associated with a modulation in the flux of glutamine to both glutamate and 2-HG. Finally, hyperpolarized ^13^C-MRS was used to show that the flux of α-KG to both glutamate and 2-HG was modulated by treatment.

**Conclusion:** In this study, we identified potential ^1^H- and ^13^C-MRS-detectable biomarkers of response to IDH1mut inhibition in gliomas. Although further studies are needed to evaluate the utility of these biomarkers *in vivo*, we expect that in addition to a ^1^H-MRS-detectable drop in 2-HG, a ^1^H-MRS-detectable increase in glutamate, as well as a hyperpolarized ^13^C-MRS-detectable change in [1-^13^C] α-KG flux, could serve as metabolic imaging biomarkers of response to treatment.

## Introduction

Recent reports estimated that over 25,000 new cases of primary malignant brain tumors would be diagnosed in the United States in 2020 1. The World Health Organization (WHO) classifies brain tumors according to their cell type (astrocytoma, oligodendroglioma and glioblastoma) and grade (I to IV) based on clinical, histological and, since the revised 2016 WHO classification, molecular/genetic criteria 1-4. Grade I tumors are typically benign, grade II and III astrocytomas and oligodendrogliomas are mostly considered low-grade gliomas (LGGs), and grade IV glioblastomas (GBMs) are classified as high-grade. Although LGGs have a significantly better prognosis than GBMs, average survival still remains below 7 years 4, 5. Standard of care for all gliomas involves maximal safe surgical resection followed by treatment, depending on grade 4. But tumor upgrade and recurrence almost always occur, leading to patient death 4, 5. Therefore, improved treatment for glioma patients is currently a major focus of the neuro-oncology community.

Up to 70-90% of LGGs and secondary upgraded GBMs harbor a mutation in the cytosolic isocitrate dehydrogenase 1 (IDH1) enzyme or, in a smaller number of cases, in the mitochondrial IDH2 6-8. The wild-type IDH enzyme catalyzes the oxidative decarboxylation of isocitrate to α-ketoglutarate (α-KG). In contrast, mutant IDH catalyzes the reduction of α-KG to 2-hydroxyglutarate (2-HG) leading to abnormal accumulation of the oncometabolite 2-HG in mutant cells. Elevated levels of 2-HG drive tumor development by leading to genome-wide histone and DNA methylation changes as well as stabilization of HIF-1α, all of which can mediate oncogenesis via multiple pathways 6-8. Therefore, the IDH mutation is now recognized as a “driver mutation” required for the initiation of LGGs, and IDH mutational status has been included as a genotypic parameter in the revised 2016 WHO classification of diffuse gliomas 3.

Due to its critical role in tumor development, targeting the IDH mutation is being considered as a potential therapeutic approach for mutant IDH gliomas 9-11. Several inhibitors have been developed 11 and are currently in clinical trials, either alone or in combination with chemotherapy or immunotherapy (e.g. clinicaltrials.gov NCT03343197, NCT02746081, NCT03030066, NCT03684811, NCT02381886). However, assessment of early response to these inhibitors using conventional non-invasive imaging techniques, such as magnetic resonance imaging (MRI) or computerized axial tomography (CT), is challenging. Both MRI and CT are mostly limited to the detection of anatomical changes, which can occur relatively late post treatment initiation. In fact, studies with other targeted inhibitors in tumors including gliomas show that treatment can lead tumor stasis rather than shrinkage 12, 13. Furthermore, LGGs are slow growing and difficult to monitor based on tumor size alone. Therefore, there is an urgent need for complementary imaging approaches which can identify early biomarkers of response to treatment and confirm target engagement prior to tumor shrinkage.

Magnetic resonance spectroscopy (MRS) is a well-established non-invasive technique that could help address the challenge of evaluating response to treatment by monitoring metabolic alterations specifically associated with mutant IDH inhibition. ^1^H-MRS informs on steady-state metabolite concentrations and ^13^C-MRS can be used complementarily to evaluate metabolic fluxes by monitoring the fate of exogenous ^13^C-labeled substrates 14-16. Furthermore, development of the hyperpolarized ^13^C-MRS technique, which uses dissolution dynamic nuclear polarization (dDNP), has been shown to enhance the signal-to-noise ratio of ^13^C-labeled metabolites by >10,000 fold 17. This provides a translational non-invasive method to evaluate real time metabolism both *in vitro* and *in vivo*, and has been successfully translated to the clinic using [1-^13^C] pyruvate as an imaging biomarker for several tumor types including glioma 18-20.

In the context of brain tumors, several studies have demonstrated the utility of MRS to differentiate tumors from normal brain and non-neoplastic lesions as well as inform on IDH mutational status 14, 21-26. ^1^H-MRS has been used to detect the oncometabolite 2-HG *in vivo* in patients with mutant IDH gliomas, and *ex vivo* in glioma biopsies 27-34. Beyond 2-HG detection, investigations of glioma cell models, tumor samples* ex vivo*, and patients *in vivo* have all demonstrated broad reprogramming of cellular metabolism that is associated with the IDH mutation 35-47. Using ^1^H-MRS, we previously investigated cells genetically engineered to express mutant IDH1 (IDH1mut) compared to their isogenic wild-type IDH1 (IDH1wt) counterparts 39. In addition to the increase in 2-HG, we observed a significant drop in intracellular steady-state levels of glutamate, phosphocholine (PC) and lactate. Using ^13^C-MRS, complementary studies showed that the drop in glutamate can be explained by a decrease in flux from ^13^C-labeled glucose that is mediated by a reduction in pyruvate dehydrogenase (PDH) activity 38, as well as a decrease in the flux from α-KG to glutamate mediated by a reduction in the activities of branched chain aminotransferase 1 (BCAT1), aspartate transaminase (AST), and glutamate dehydrogenase (GDH) 47. Additionally, the changes in PC have been linked to several alterations in lipid metabolism 45, 46. The ^1^H- and ^13^C-MRS findings have also been leveraged to develop hyperpolarized ^13^C-MRS approaches to image IDH1 status. In particular, the reduced flux to glutamate can be imaged using hyperpolarized [2-^13^C] pyruvate as well as hyperpolarized [1-^13^C] α-KG 38, 47. Hyperpolarized [1-^13^C] α-KG can also be used to image the increased flux to 2-HG 35. Collectively, these studies identified an MRS-detectable metabolic signature associated with the IDH1 mutation.

Based on the above-mentioned findings, the goal of this investigation was to examine the hypothesis that our previously identified ^1^H- and ^13^C-MRS-detectable metabolic alterations would be reversed with mutant IDH1 inhibition and thus serve as biomarkers for assessing the effect of, and potential response to, mutant IDH inhibitors. Our studies used two orally available small molecule inhibitors which are currently in clinical trials 9. AG-120 is a potent first-in-class IDH1mut inhibitor 48, while AG-881 is a potent first-in-class, brain penetrant inhibitor of both IDH1mut and IDH2mut 49. We investigated their intracellular metabolic effects on two genetically engineered IDH1mut-expressing cell lines using ^1^H-, ^13^C-, and hyperpolarized ^13^C-MRS 35, 38, 39, 47 and observed that some, but unexpectedly not all, of our previously identified IDH1mut-associated intracellular metabolic alterations were reversed with treatment. Our studies demonstrate that IDH1mut inhibition induces a unique metabolic profile which is detectable using clinically translatable MRS metabolic imaging and could potentially improve the monitoring of IDH1mut inhibitor treatment for glioma patients.

## Materials and Methods

### Cell culture and drug treatment

NHA and U87 cell lines expressing the mutant gene IDH R132H (NHAIDH1mut and U87IDH1mut), and an NHA cell line expressing wild-type IDH1 (NHAIDH1wt), were generated and maintained in Dulbecco's Modified Eagle Medium (DMEM; Gibco, USA) supplemented with 10% fetal calf serum, 2 mM glutamine, and 100 U/mL penicillin and streptomycin under normoxic conditions as previously described 39. Both cell lines were routinely tested for mycoplasma contamination and authenticated by short tandem repeat fingerprinting (Cell Line Genetics, USA) within 6 months of any study.

AG-881 (MedChemExpress, USA) and AG-120 (MedChemExpress, USA) solutions (1000X) were prepared by mixing drug powder with DMSO (Sigma Aldrich, USA). For all experiments, treatment with 1 µM AG-881, 1 µM AG-120, or DMSO (vehicle, 0.1%) began one day after seeding, when cells had adhered to flasks, and cells were treated every 24 h for 72 h. Drug dosages were based on previous publication with a biosimilar 50 and confirmed in our cell models.

### Spectrophotometric enzyme assays

All spectrophotometric measurements were performed on an Infinite m200 spectrophotometer (Tecan Systems, Inc., USA). NHAIDH1mut and U87IDH1mut cells were grown and treated as described above, collected, and samples prepared according to manufacturer instructions to quantify enzyme activities.

IDH1mut activity was quantified using a colorimetric assay kit (BioVision, USA). Briefly, ~1.0 × 10^7^ cells were lysed in kit buffer and 25 µL of sample was loaded per well with reaction mix. Absorbance at 450 nm, reflecting NADPH consumption, was measured every 1 min for 1 h at 37 ºC. Enzyme activity was calculated by converting absorbance/min into nmol/min using an NADPH standard and normalizing to cell number as per manufacturer instructions.

Glutamate dehydrogenase (GDH) and aspartate transaminase (AST) activities were measured using assay kits (Abcam, UK). For both assays, ~1.0 × 10^7^ cells were lysed in kit buffer and 50 µL of sample loaded per well. Absorbance at 450 nm was measured every 1 min for 1 h at 37 ºC. Activities of GDH and AST were calculated using standard curves of NADH and glutamate, respectively, and expressed in fmol of NADH/cell/min or fmol of glutamate/cell/min.

The BCAT1 activity assay replicated methods described by Chaumeil *et al.* 47. Briefly, ~1.0 × 10^7^ cells were lysed and 20 µL of lysate loaded per well. Absorbance was measured at 340 nm every 30 s for 10 min and reaction rate was calculated for each sample and expressed as fmol NADH/cell/min.

### Cell proliferation and clonogenicity

Impact of treatment on cell proliferation was determined by counting the number of cells at the time of extraction. Cells were aliquoted and counted using a Cellometer Mini Automated Cell Counter (Nexcelom Bioscience, USA). Clonogenicity of control and treated NHAIDH1mut and U87IDH1mut cells was determined by seeding ~500 cells per well in 6-well plates containing 2 mL DMEM (Gibco, USA), similar to previous studies 51. After 24 h, media was replaced and treatment initiated. Cells were treated every 24 h until colonies could be observed (10-15 days depending on cell line) at which point colonies were gently washed, incubated at room temperature with 0.01% crystal violet (Sigma-Aldrich, USA; ref. HT90132-1L) for 30 min and stained colonies were counted.

### ^1^H-MRS of cell extracts

Control and treated NHAIDH1mut, U87IDH1mut, and NHAIDH1wt cells were extracted using the dual-phase extraction method as previously described 52. Briefly, ~3.0 × 10^7^ cells were trypsinized, centrifuged and vortexed in 10 mL of ice-cold methanol followed by 10 mL each of ice-cold chloroform and ice-cold water. Following phase separation, the aqueous phase was lyophilized and resuspended in 400 μL of deuterium oxide (Cambridge Isotope Laboratories, USA). 5 mM sodium 3-(trimethylsilyl)propionate-2,2,3,3-d4 (TSP) (Cambridge Isotope Laboratories, USA) was placed in a coaxial insert and used as an external chemical shift and quantification reference. Aqueous phase spectra (90° flip angle (FA), 3 s repetition time (TR), 384 scans) were acquired using a 500 MHz Bruker Avance spectrometer (Bruker Biospin, Germany). Prior to Fourier transformation, a line broadening of 0.3 Hz was applied to FIDs. All detectable metabolites were then quantified by peak integration following deconvolution of overlapping peaks using Mnova (MestreLab Research, Spain). Peak integrals were corrected for saturation and Nuclear Overhauser effect (NOE) correction factors and normalized to cell number as previously published 39.

### ^13^C-MRS of cell extracts

NHAIDH1mut and U87IDH1mut cells were grown in media in which glucose and glutamine were replaced with [1-^13^C] glucose (5.5 mM) or [3-^13^C] glutamine (2.2 mM), supplemented with ^12^C glucose and ^12^C glutamine to reach normal concentration (25 mM glucose; 6 mM glutamine), and treated as described above. Approximately 1.0 × 10^8^ cells were extracted by dual-phase extraction 52 and prepared as described above. Similarly to our previous studies 38, ^13^C spectra were acquired with a standard Bruker 1D proton-decoupled ^13^C sequence (zgpg30) using a 500 MHz Bruker Avance spectrometer (Bruker Biospin, Germany). Spectra were acquired with a 30° flip angle and 3 second relaxation delay with a total of 4000 averages. In addition, a fully relaxed spectrum (90° flip angle, 60 second relaxation delay and broad-band decoupling applied during the acquisition time only) was acquired and used to estimate the saturation and NOE correction factors. Prior to Fourier transformation, a line broadening of 1.0 Hz was applied. All spectral assignments were based on literature reports and quantification of peak integrals was performed after deconvolution of overlapping peaks using Mnova (Mestrelab Research, Spain). Data were then corrected for saturation and NOE, and normalized to an external reference of known concentration (43 mM TSP) and cell number.

### Hyperpolarized ^13^C-MRS

#### Hyperpolarized [2-^13^C] pyruvate studies

6 µL of [2-^13^C] pyruvate solution (14.4 M [2-^13^C] pyruvate, 19.5 mM OX63 radical, 1.5 mM Dotarem) was polarized using a Hypersense DNP polarizer (Oxford Instruments, UK) for approximately 1 h similar to previous publications 38 (based on previous work we expect ~20-30% polarization ratio 53). Hyperpolarized [2-^13^C] pyruvate was then rapidly dissolved in 6 mL Tris-based isotonic buffer and 300 µL was injected into a suspension of ~5.0 × 10^7^ live cells to a final concentration of 5 mM. Dynamic sets of ^13^C spectra were immediately acquired using a 1.5 Tesla Pulsar (Oxford Instruments, UK) spectrometer (20º FA, 3 s TR, 100 scans).

#### Hyperpolarized [1-^13^C] α-KG studies

30 µL of [1-^13^C] α-KG solution (5.9 M [1-^13^C] α-KG, 3:1 water:glycerol, 17.3 mM OX63 radical, 0.4 mM Dotarem) was polarized using a Hypersense polarizer (Oxford Instruments, UK) for approximately 1.5 h as previously described 35, 47 (based on previous work we expect ~15% polarization ratio 35). Hyperpolarized [1-^13^C] α-KG was rapidly dissolved in 5 mL Tris-based isotonic buffer and 300 µL was injected into a suspension of ~5.0 × 10^7^ live cells to a final concentration of 15 mM. Dynamic sets of ^13^C spectra were immediately acquired using a 1.5 Tesla Pulsar (Oxford Instruments, UK) spectrometer (20º FA, 3 s TR, 100 scans). In addition, to clearly distinguish the [1-^13^C] 2-HG product peak from that of the natural abundance [5-^13^C] α-KG, hyperpolarized [1-^13^C] α-KG was injected into cell lysates as previously described 35. Briefly ~1.5 × 10^8^ cells were lysed, placed in a 5 mm NMR tube and mixed with 1 mM each of NADPH and NADH immediately prior to injection of ~500 µL hyperpolarized [1-^13^C] α-KG solution (15 mM final concentration). Dynamic sets of ^13^C spectra were immediately acquired using an 11.7 Tesla (Agilent Technologies, USA) spectrometer (13º FA, 3 s TR, 100 scans).

^13^C spectra were analyzed using Mnova (MestreLab Research, Spain). For dynamic analysis, metabolites were quantified by peak integration for each time point, then normalized to the maximum substrate peak ([2-^13^C] pyruvate or [1-^13^C] α-KG) and cell number as previously described 35, 38, 47, 54. We also quantified the area under the curve for product and normalized to substrate and cell number.

### Statistical analysis

All experiments were performed at least 3 times unless otherwise stated and results are expressed as mean ± standard deviation. An unpaired Student's *t*-test assuming unequal variance with *p* < 0.05 considered significant was used to compare findings with a statistical power ≥90% except for PC for which power was >80% (*p < 0.05, **p < 0.01, ***p < 0.001 and n.s. not significant). ^1^H-MRS data was also analyzed using One-Way ANOVA test with multiple comparison (using Bonferroni correction) (Table S1, S2, and S3).

## Results

**Figure 1:**
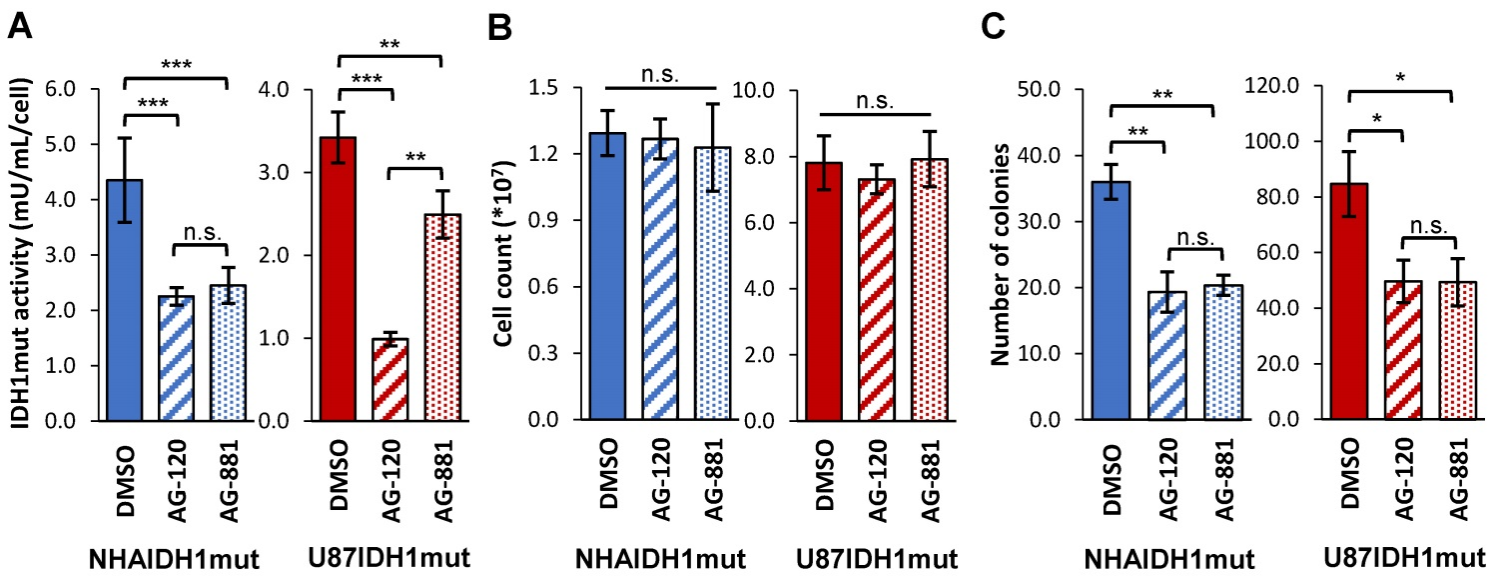
** IDH1mut enzyme activity and clonogenicity are affected by IDH1mut-targeted treatment. (A)** IDH1mut enzyme activity was reduced after both treatments in both NHA (blue) and U87 (red) IDH1mut cells. **(B)** Cell number was not affected by IDH1mut inhibitors. **(C)** Clonogenicity assays show significant decrease in number of colonies following both treatments. IDH1mut: mutant isocitrate dehydrogenase; n.s.: not significant.

Our studies were performed on two genetically engineered IDH1mut-expressing cell models, NHAIDH1mut and U87IDH1mut. Prior to investigating the metabolic effects of AG-120 and AG-881, we first confirmed the activity of these drugs on our cell models by looking at IDH1mut enzyme activity, cell number, and clonogenicity. As illustrated in Figure 1A, treatment of NHAIDH1mut cells with 1 µM AG-120 (n = 3) or 1 µM AG-881 (n = 3) resulted in a significant 48% drop (*p* < 0.001) and 44% drop (*p* < 0.001) respectively in IDH1mut activity, compared to DMSO-treated control cells (n = 9), consistent with a recent publication regarding the enzyme IC_50_ and inhibitor efficacy at 3-5 times the IC_50_ 49. There was no significant change in cell number after treatment with either inhibitor (n = 6) (Figure 1B), indicating that treatment did not alter cell proliferation or result in cell death. However, the inhibition of IDH1mut activity was associated with a significant drop in the number of colonies produced by cells treated with AG-120 and AG-881 by 46% (*p* < 0.01) and by 44% (*p* < 0.01), respectively, compared to control cells (n = 3) (Figure 1C). We also confirmed our results in U87IDH1mut cells. Similar to the NHA model, IDH1mut activity was significantly decreased by treatment with AG-120 (n = 4) (71%, *p* < 0.001) and AG-881 (n = 4) (27%, *p* < 0.01) (Figure 1A). Treatment with IDH1mut inhibitors did not cause a significant change in cell number (n = 6) (Figure 1B). Additionally, there was a significant drop (41%, *p* < 0.05; and 42%, *p* < 0.05) in the number of colonies produced by U87IDH1mut cells treated AG-120 and AG-881, respectively (n = 3 for each) (Figure 1C).

**Figure 2:**
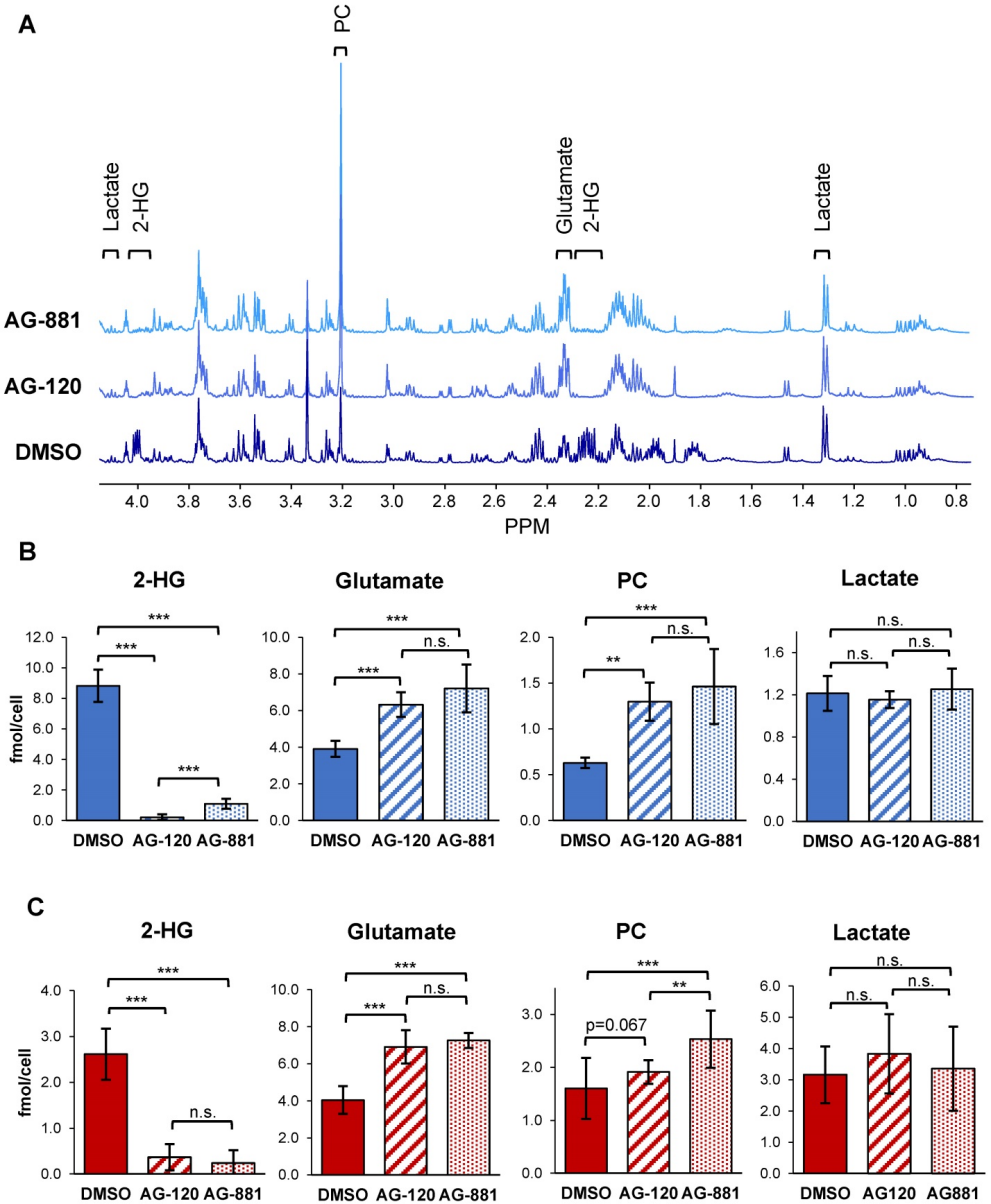
**^1^H-MRS spectra show AG-120 and AG-881 affect intracellular steady-state metabolite levels in IDH1mut cells. (A)** Representative ^1^H-MRS spectra of NHAIDH1mut cells treated with DMSO, (bottom), AG-120 (center), and AG-881 (top). **(B)** Quantification of steady-state NHAIDH1mut metabolite concentrations. Results illustrate that AG-120 and AG-881 affect 2-HG, PC and glutamate levels, but not lactate. **(C)** Quantification of steady-state metabolites in U87IDH1mut cells, supporting NHAIDH1mut results. 2-HG: 2-hydroxyglutarate; IDH1mut: mutant isocitrate dehydrogenase; n.s.: not significant; PC: phosphocholine.

To test the hypothesis that IDH1mut inhibition reverts the MRS-detectable metabolic reprogramming associated with the IDH1 mutation, we used ^1^H-MRS and quantified steady-state metabolite levels in control and treated NHAIDH1mut (n = 9/5/8 DMSO/AG-120/AG-881) and U87IDH1mut (n = 16/11/11 DMSO/AG-120/AG-881) cells (Table S1, S2 and Figure 2). As expected, our results showed that treatment of NHAIDH1mut cells with AG-120 or AG-881 led to a significant 98% decrease (*p* < 0.001) and 88% decrease (*p* < 0.001) in steady-state 2-HG levels, respectively (Figure 2B). In addition, we detected a significant increase in glutamate levels (AG-120: 62%, *p* < 0.001; AG-881: 85%, *p* < 0.001) compared with DMSO-treated controls. PC levels also increased (AG-120: 106%, *p* < 0.01; AG-881: 133%, *p* < 0.001). However, counter to our expectation, intracellular lactate levels, which were reported to be modulated by the IDH1 mutation, were not significantly altered by treatment (AG-120: *p* = 0.85, AG-881: *p* = 0.69). None of the other MR-detectable metabolites previously investigated were altered either (Table S1) 39. We confirmed our results in U87IDH1mut cells (Table S2 and Figure 2C) and found that treatment with IDH1mut inhibitors also led to a significant decrease in 2-HG (AG-120: 89%, *p* < 0.001; AG-881: 91%, *p* < 0.001). We also detected a significant increase in steady-state glutamate levels (AG-120: 71%, *p* < 0.001; AG-881: 80%, *p* < 0.001). However, the increase in PC only trended to significance with AG-120 (19%, *p* = 0.067), although it was significant with AG-881 treatment (58%, *p* < 0.001). Also, similarly to NHAIDH1mut cells and counter to our expectation, no significant changes in intracellular lactate levels were observed (AG-120: *p* = 0.15, AG-881: *p* = 0.67).

To confirm that these metabolic alterations resulted from inhibition of the IDH1mut enzyme specifically, we also examined ^1^H-MRS-detectable metabolite levels in control and treated NHAIDH1wt cells (n = 3 for each). Unlike the IDH1mut models, we did not observe any significant metabolic alterations after treatment of NHAIDH1wt cells with AG-120 or AG-881, compared with DMSO-treated control cells (Table S3 and Figure S1).

**Figure 3:**
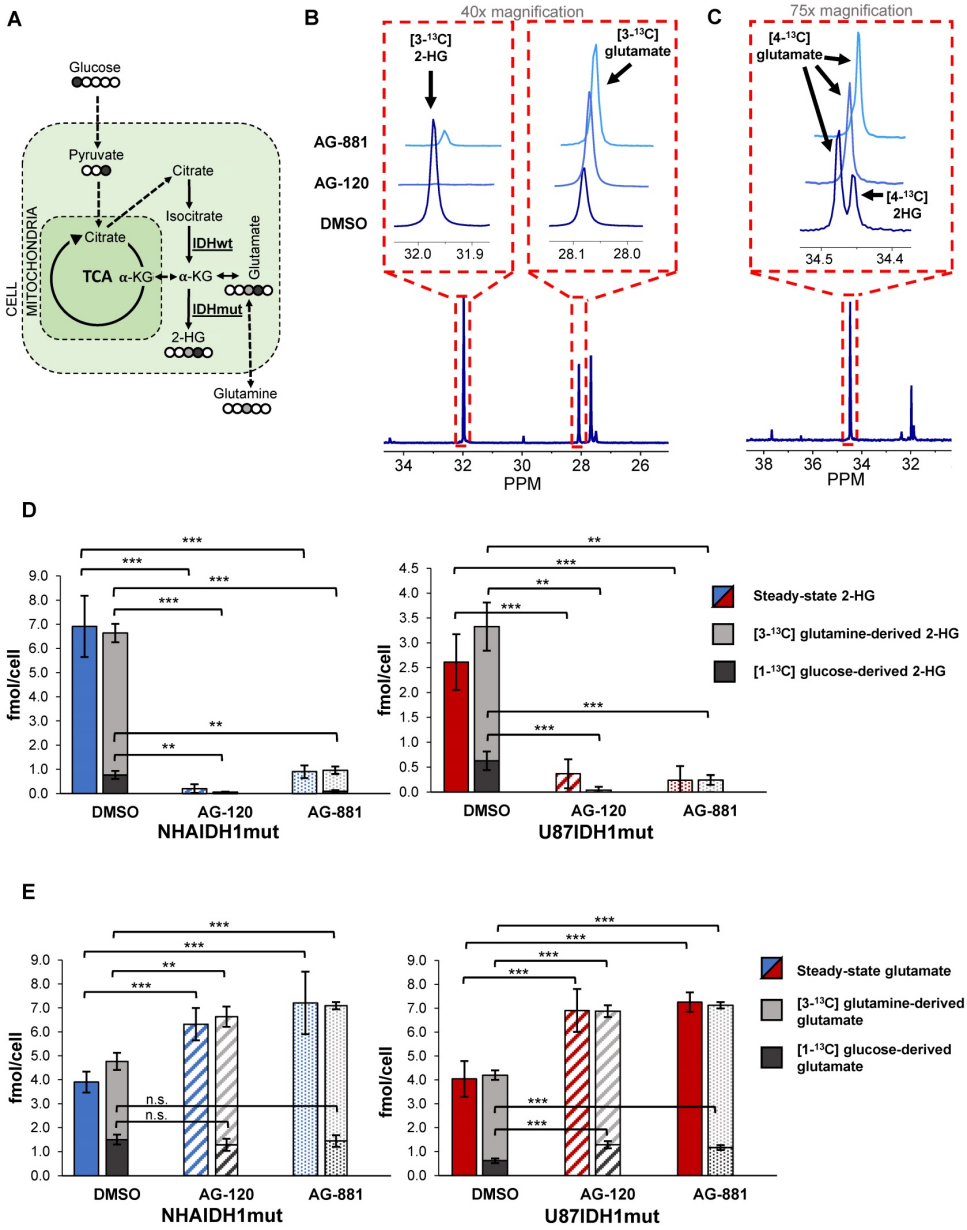
** Changes in 2-HG and glutamate steady-state levels are associated with changes in metabolic fluxes. (A)** Metabolic pathway showing ^13^C labeling of glutamate and 2-HG from [1-^13^C] glucose and [3-^13^C] glutamine. **(B)** Representative ^13^C-MRS spectra of NHAIDH1mut cells labeled with [3-^13^C] glutamine and **(C)** labeled with [1-^13^C] glucose (regions of labeled glutamate and 2-HG peaks expanded) **(D)** Quantification of 2-HG peaks produced from [1-^13^C] glucose and [3-^13^C] glutamine (measured from ^13^C-MRS data) and total 2-HG levels (measured from ^1^H-MRS data) shows decreased metabolic flux from both [1-^13^C] glucose and [3-^13^C] glutamine to 2-HG in both NHA (blue) and U87 (red) IDH1mut cells. **(E)** Quantification of ^13^C glutamate peaks from ^13^C-MRS and ^1^H-MRS data shows increased metabolic flux from [3-^13^C] glutamine to glutamate in both cell lines. There is also an increase in flux from [1-^13^C] glucose to glutamate in U87IDH1mut cells (red) but not NHAIDH1mut cells (blue). 2-HG: 2-hydroxyglutarate; IDH1mut: mutant isocitrate dehydrogenase; IDHwt: wild-type isocitrate dehydrogenase; n.s.: not significant.

To further confirm our findings, and assess whether hyperpolarized probes previously shown to be informative of IDH1 status 35, 38, 47 could also be used to probe IDH1 inhibition, we next examined how treatment-induced changes in intracellular 2-HG and glutamate were associated with altered metabolic fluxes. To that end, we probed 2-HG and glutamate synthesis from extracellular [1-^13^C] glucose and [3-^13^C] glutamine, the main precursors of these metabolites 38 (Figure 3A). Using ^13^C-MRS, we quantified intracellular [3-^13^C] 2-HG and [3-^13^C] glutamate (derived from [3-^13^C] glutamine) (NHAIDH1mut: n = 7/3/4; U87IDH1mut: n = 4/3/5 DMSO/AG-120/AG-881) (Figure 3B) as well as [4-^13^C] 2-HG and [4-^13^C] glutamate (derived from [1-^13^C] glucose) (NHAIDH1mut: n = 8/3/5; U87IDH1mut: n = 8/4/5 DMSO/AG-120/AG-881) (Figure 3C) and compared the contributions from glucose and glutamine to the total steady-state 2-HG and glutamate pools. In NHAIDH1mut cells, our results showed a significant decrease in the flux to 2-HG from both [1-^13^C] glucose (AG-120: 100%, *p* < 0.001; AG-881: 88%, *p* < 0.001) and [3-^13^C] glutamine (AG-120: 99%, *p* < 0.001; AG-881: 85%, *p* < 0.001) (Figure 3D). In contrast, when considering glutamate, flux from [3-^13^C] glutamine to glutamate significantly increased in cells treated with AG-120 (64%, *p* < 0.001) or AG-881 (73%, *p* < 0.001) while flux from [1-^13^C] glucose to glutamate was unchanged (*p* > 0.05) (Figure 3E). Findings in U87IDH1mut cells were similar. We observed a significant drop in the flux to 2-HG from both [1-^13^C] glucose (AG-120 and AG-881: 100%, *p* < 0.001) and [3-^13^C] glutamine (AG-120: 99%, *p* < 0.01; AG-881: 88%, *p* < 0.01) (Figure 3D). Unlike the NHAIDH1mut cells, we observed a significant increase in the flux from [1-^13^C] glucose to glutamate (AG-120: 74%, *p* < 0.001; AG-881: 88%, *p* < 0.001). However, similar to NHAIDH1mut, we also observed a significant increase in the flux of [3-^13^C] glutamine to glutamate in AG-120 (57%, *p* < 0.001) and AG-881 (67%, *p* < 0.001) treated cells (Figure 3E).

In light of these changes in metabolic fluxes, we next considered the likely utility of hyperpolarized ^13^C-MRS as a clinically translatable imaging approach to assess response to therapy in glioma and focused on investigating the more clinically relevant brain-penetrant inhibitor AG-881 [49 and http://investor.agios.com/]. Our ^13^C-MRS results showed no change in flux from [1-^13^C] glucose to glutamate following IDH1mut inhibition in NHAIDH1mut cells (Figure 3E). This indicated that hyperpolarized [2-^13^C] pyruvate would likely not be useful to probe the production of glutamate in our study. We confirmed this hypothesis in a small scale study in the NHAIDH1mut cells (n = 2) where we did not observe a change in flux from [2-^13^C] pyruvate to [5-^13^C] glutamate when comparing control (5.75±0.92 AU/ cell) and AG-881 treated (6.25±1.04 AU/cell) cells (Figure S2A-B). There was also no change in [2-^13^C] pyruvate flux to [2-^13^C] lactate after AG-881 treatment (from 8.58±0.43 to 7.56±0.87 AU/cell) (Figure S2C). Since our goal was to identify biomarkers common to both our cell models, we did not investigate pyruvate any further.

Next, we considered the utility of hyperpolarized [1-^13^C] α-KG for detecting the effect of IDH1mut inhibition. Based on our previous studies showing that hyperpolarized [1-^13^C] α-KG can detect elevated 2-HG synthesis and decreased glutamate synthesis in IDH1mut cells and tumors 35, 47, we hypothesized that this approach could detect a drop in 2-HG and increase in glutamate production following IDH1mut inhibition. We had also shown that the reduced conversion from α-KG to glutamate in mutant IDH1 tumors is mediated by a drop in GDH, BCAT1, and AST activities 47. Therefore, in support of our hypothesis, we examined the activity of GDH, BCAT1, and AST, and detected a significant increase in the activity of GDH (30%, *p* < 0.05) in NHAIDH1mut cells treated with AG-881 (Figure S3A), but did not detect any changes in BCAT1 and AST activity (Figure S3B). Based on these findings we next investigated the metabolism of hyperpolarized [1-^13^C] α-KG to both glutamate and 2-HG.

**Figure 4:**
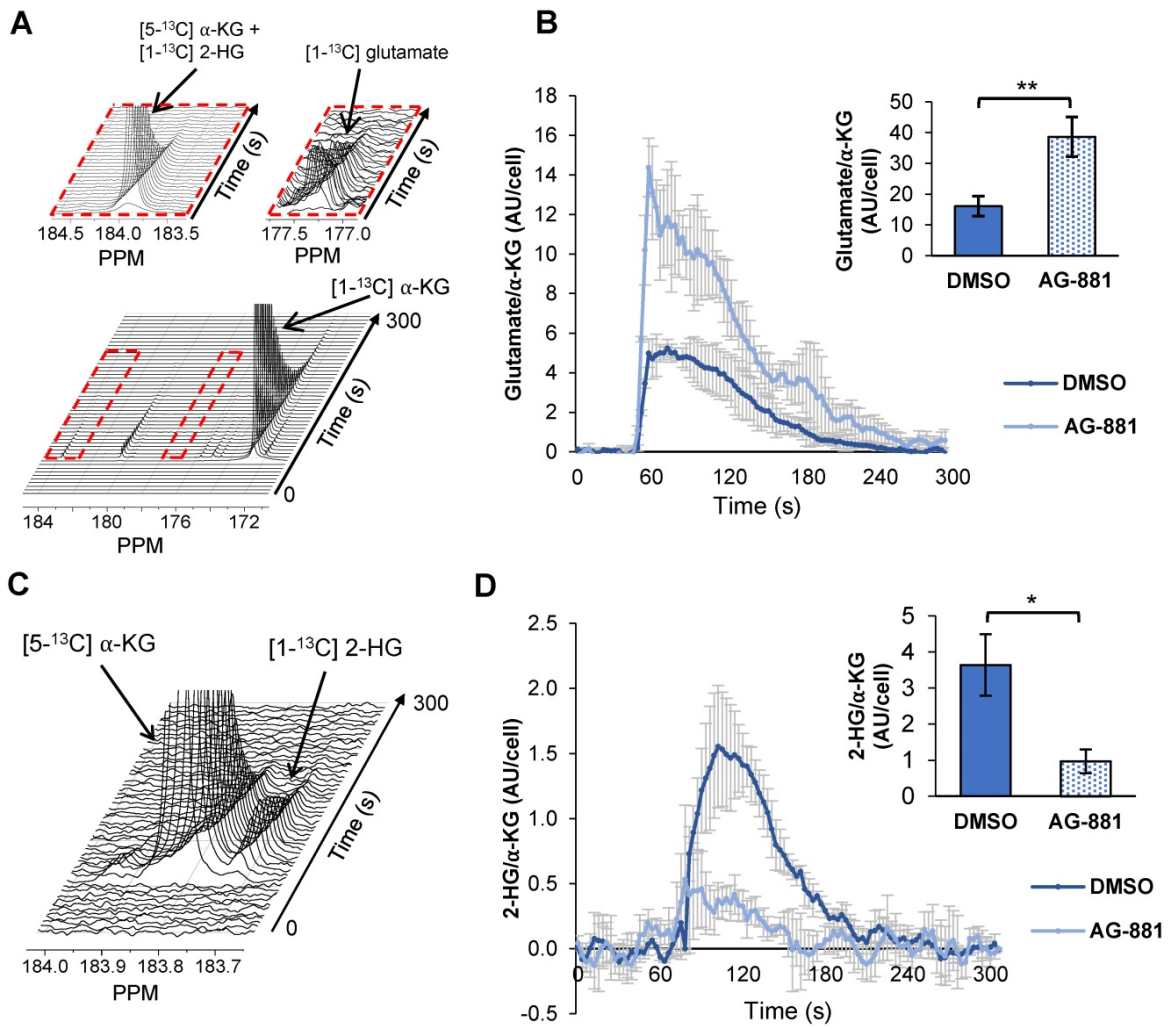
** Fluxes from hyperpolarized [1-^13^C] α-KG to [1-^13^C] glutamate and [1-^13^C] 2-HG are altered after AG-881-treatment in NHAIDH1mut cells. (A)** Representative ^13^C-MRS spectral array of [1-^13^C] glutamate production from hyperpolarized [1-^13^C] α-KG in live NHAIDH1mut cells acquired at 1.5 Tesla (region of [1-^13^C] glutamate and [5-^13^C] α-KG/[1-^13^C] 2-HG peaks expanded; [5-^13^C] α-KG and [1-^13^C] 2-HG peaks overlap). **(B)** Quantification of ^13^C-MRS spectra of NHAIDH1mut cells shows increased [1-^13^C] glutamate production following treatment. **(C)** ^13^C-MRS acquisition of hyperpolarized [1-^13^C] α-KG injection into NHAIDH1mut cell lysates at 11.7 Tesla allows for clear resolution of [1-^13^C] 2-HG from [5-^13^C] α-KG peak. **(D)** Quantification of 11.7 T spectra shows decreased 2-HG production following AG-881 treatment. 2-HG: 2-hydroxyglutarate; α-KG: alpha-ketoglutarate.

**Figure 5:**
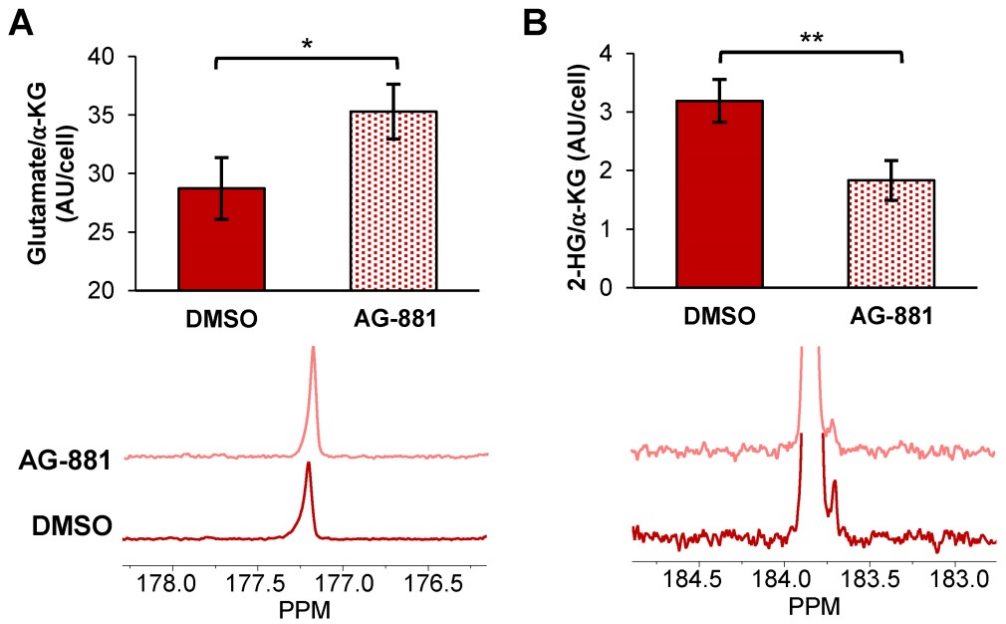
** Fluxes from hyperpolarized [1-^13^C] α-KG to [1-^13^C] glutamate and [1-^13^C] 2-HG are altered after AG-881 treatment in U87IDH1mut cells (A)** Quantification (top) of summed ^13^C-MRS spectra (bottom) of live U87IDH1mut cells at 1.5 T shows increased [1-^13^C] glutamate production from hyperpolarized [1-^13^C] α-KG following treatment. **(B)** Quantification (top) of summed^ 13^C-MRS spectra (bottom) of U87DHmut cell lysates at 11.7 T shows decreased [1-^13^C] 2-HG production from hyperpolarized [1-^13^C] α-KG following AG-881 treatment. 2-HG: 2-hydroxyglutarate; α-KG: alpha-ketoglutarate.

Injection of hyperpolarized [1-^13^C] α-KG into control NHAIDH1mut cells resulted in a detectable buildup of glutamate as previously published 47 (Figure 4A) and, importantly, cells treated with AG-881 (n = 3) showed significantly higher flux from α-KG to glutamate compared with DMSO-treated control cells (n = 3; increasing 141%, *p* < 0.05) (Figure 4B). Investigating cell lysates allowed clear resolution of 2-HG from the naturally abundant [5-^13^C] α-KG, which resonate at 183.9 ppm and 184 ppm respectively 35 (Figure 4C), and demonstrated a detectable drop in α-KG flux to 2-HG in treated cells (decreasing 67%, *p* < 0.01) (Figure 4D). Findings in U87IDH1mut were similar to NHAIDH1mut. Flux from hyperpolarized [1-^13^C] α-KG to glutamate significantly increased in AG-881-treated U87IDH1mut cells (23%, *p* < 0.05) (Figure 5A) and flux to 2-HG was significantly reduced (43%, *p* < 0.01) (Figure 5B). Collectively, our data indicate that altered flux to both 2-HG and glutamate can be detected using hyperpolarized [1-^13^C] α-KG.

## Discussion

The IDH1 mutation is found in over 70-90% of LGGs and upgraded GBMs and its inhibition is therefore an attractive therapeutic approach. Targeted inhibitors of IDH1 and pan-IDH1/2 mutations, such as AG-120 and AG-881 respectively, are in clinical trials and have shown promising results [9-11 and http://investor.agios.com/]. Clinical trials with AG-120 have shown substantial 2-HG reduction and disease control in patients with advanced tumors including acute myeloid leukemia (AML) and chondrosarcoma (e.g. clinicaltrials.gov NCT02073994 and NCT02074839) 11, 55, and AG-120 continues to be investigated either alone or in combination with traditional chemotherapies (e.g. clinicaltrials.gov NCT03245424 and NCT03173248). When considering gliomas, AG-120 has shown 2-HG suppression but low brain penetrance in preclinical models indicating that it may not effectively cross the blood brain barrier to treat glioma patients 48. Thus, it was recently announced that AG-120 will not be moving forward independently in clinical trials for glioma [http://investor.agios.com/]. Nevertheless, the biomarkers we identified in this study could potentially be useful for response assessment in those tumor types which are responsive to AG-120, albeit after further studies to confirm the generality of our findings. AG-881 on the other hand, has shown adequate brain penetration, significantly decreases 2-HG accumulation in gliomas as detected by mass spectrometric measurements of tumor biopsies, and demonstrates promising clinical activity in early clinical trials (e.g. clinicaltrals.gov NCT02481154 and NCT03343197) 49, 56. As a result, a phase 3 multicenter clinical study using AG-881 in glioma patients was recently initiated and will provide further insight into the efficacy of this novel therapeutic (clinicaltrials.gov NCT04164901).

Importantly however, *in vivo* studies with AG-881 and other mutant IDH inhibitors have shown that although treatment inhibits tumor growth, reduces cell density 57, and may extend patient survival, there is no evidence of clearly detectable tumor shrinkage [9 and http://investor.agios.com/]. Consistent with this observation, we found that treatment of our cell models with both AG-120 and AG-881 significantly inhibited IDH1mut enzyme activity and led to a 50% drop in clonogenicity but did not affect cell numbers indicating no cell death or inhibition of cell proliferation (Figure 1). These observations appear consistent with the complex, and yet to be fully understood, process through which inhibition of mutant IDH suppresses tumor growth but may not lead to readily detectable tumor shrinkage *in vivo* 9. Accordingly, conventional anatomic imaging methods may not effectively assess response to treatment. This highlights the need for early biomarkers of drug delivery, drug action, and likely response to IDHmut inhibition, and our study identifies such potential biomarkers.

Our studies were performed in two genetically engineered cell models. The NHAIDH1mut model, which was generated from immortalized normal human astrocytes 39, 58, provides a robust platform to investigate the role and consequences of mutant IDH1 but is unlikely to reproduce the full complexity of a mutant IDH1 tumor in the patient. In contrast, the U87IDH1mut model was generated from patient U87 glioblastoma cells and, as such, likely harbors oncogenic mutations associated with the clinical development of GBM but not of LGG 39. The different backgrounds of our models possibly explain some of the small metabolic differences observed in these cell lines following treatment (e.g. there is a change in glucose flux to glutamate for U87IDH1mut but not for NHAIDH1mut). Indeed, small differences in mutant IDH1-associated metabolism have been previously reported in these cell models 12, 38, 39. It should also be noted that we did not investigate a model of mutant IDH2, which is a much less common mutation in glioma. In spite of the limitations of our models, this investigation identified common metabolic alterations associated with IDH1mut inhibition in both our cell lines. We have previously observed that IDH1-associated biomarkers identified in cells are also detected in orthotopic brain tumors *in vivo* 12, 35, 47. This study therefore sets the scene for future investigations in patient-derived orthotopic tumor models *in vivo*, and ultimately in the clinical setting. Such studies will be essential to assess the value of our findings as translatable metabolic biomarkers of response to therapy.

As mentioned, previous studies in our lab found that genetically engineered IDH1mut cells that differ from IDH1wt cells only in their IDH1 status, have a unique ^1^H-MRS-detectable metabolic signature distinguished by an increase in steady-state 2-HG, and a decrease in glutamate, PC, and lactate 39 in agreement with other cell studies 42, 59, 60. *In vivo* studies comparing mutant IDH glioma biopsies to wild-type IDH GBM biopsies also showed lower glutamate levels, in spite of the vastly disparate genetic landscape of these tumor types, but trends in other metabolites were more complex 40. Additionally, studies have also shown that glutamate levels are lower in mutant IDH tumors compared to normal brain 61. When assessing the effect of mutant IDH inhibitors in our cells, we observed that IDH1mut inhibition led to the expected decrease in steady-state 2-HG, in line with prior *in vitro*, *in vivo*, and *ex vivo* studies with AG-120 and AG-881 9-11, 48, 49, 55, 62-64. Furthermore, and consistent with our hypothesis that the IDH1mut biomarkers would be reversed with treatment, we also observed a significant increase in steady-state glutamate in both NHAIDH1mut and U87IDH1mut cells. However, counter to our hypothesis, there was no change in lactate in either cell line and there was only a trend to an increase in PC when considering both cell lines and both inhibitors. Taken together, our data thus shows that IDH1mut inhibition leads to a modulation of some, but not all, of the metabolites previously shown to be affected by the IDH1 mutation. This indicates that not all the molecular events downstream of 2-HG are reversed by its treatment-induced reduction and suggests that IDH1mut inhibition induces a distinct ^1^H-MRS-detectable metabolic profile. Further studies are needed to fully understand the mechanistic underpinnings of these metabolic alterations and investigate any potential correlations between 2-HG and the other MR-detectable changes.

In the clinical setting, several groups have used ^1^H-MRS to detect 2-HG, glutamate, lactate, and choline-containing metabolites (tCho, which includes choline, PC, and glycerophosphocholine) 27-34, 40, 65. Indeed, ^1^H-MRS is currently incorporated into ongoing clinical trials using IDH1mut inhibitors 57 and, importantly, the ^1^H-MRS detectable metabolic changes observed in the clinic are consistent with our observations 57. Most notably, a significant drop in 2-HG, and a trend to an inverse correlation between the changes in 2-HG and the changes in the composite glutamate plus glutamine peak (Glx), were observed following treatment with the mutant IDH1 inhibitor IDH305 57.

It should be noted that quantifying 2-HG requires the use of specifically optimized MRS sequences due its proximity to glutamine and glutamate 27-34. Similarly, glutamate is reliably quantified at 7 Tesla, but at lower clinical fields strengths (1.5 and 3 Tesla) it resonates close to, and is typically measured together with, glutamine as Glx, or quantified through the use of a dedicated optimized sequence 57, 61, 66. Thus, care should be taken in acquiring and interpreting the clinical data. Nonetheless, in our study, no changes in intracellular steady-state glutamine levels were detected, suggesting that monitoring Glx at a lower magnetic field could offer an indirect reading of changes in glutamate levels and confirm the effects of treatment.

Another metabolic change reported in the clinical study 57 was a trend to a small 9% increase in lactate. Our data in cells did not identify a significant change in lactate levels. However, it is important to note that our data reflects intracellular lactate levels only, whereas the* in vivo* patient data captures both the intra- and extracellular compartments. Thus, the trend towards a small increase in lactate in patients could be indicative of changes in extracellular lactate accumulation.

Similarly, the discrepancy between our cellular PC data, which trended to an increase, and the early clinical observation that tCho (choline, PC, and glycerophosphocholine) is not significantly altered in treated patients, is likely reflective of the complexity of factors that modulate tCho levels, especially in the *in vivo* clinical setting. On one hand, an increase in intracellular tCho levels can be due to oncogenic mutations, or as shown in this study, response to mutant IDH inhibitors 67-69. On the other hand, tCho levels are associated with tumor cellularity and will therefore drop when cell numbers drop in response to treatment 67, 70. The clinical study by Andronesi *et al.* showed that treatment with a mutant IDH inhibitor led to an increase in diffusion, indicating a decrease in cellular density 57. Since the *in vivo* tCho signal reflects both cell density *and* intracellular metabolite levels, any potential increase in intracellular PC levels might therefore not be detectable because of the overall reduction in cell number*.* This highlights the likely limitation of PC and tCho as *in vivo* biomarkers for monitoring response to mutant IDH inhibitors.

A complementary method to ^1^H-MRS, which probes steady-state metabolite levels, is hyperpolarized ^13^C-MRS, which provides a tool to monitor metabolic fluxes in real-time. Hyperpolarized ^13^C-MRS requires both additional expertise and equipment that is currently only available in large research and medical centers. Nonetheless, over the past decade, several hyperpolarized ^13^C agents have been developed and applied to the imaging of normal and diseased cells, animal models and patients *in vivo* 18-20, 35, 47, 71-73. Most notably, hyperpolarized [1-^13^C] pyruvate and [2-^13^C] pyruvate are currently in clinical trials for glioma patients 20, 74. Additionally, multiple earlier studies with different chemotherapeutic agents and targeted therapies have used hyperpolarized [1-^13^C] pyruvate in animal models as well as in prostate cancer patients and have shown that a drop in its flux to lactate is associated with response to therapy 19, 75-79. However, as shown in this study, the utility of pyruvate to assess response to mutant IDH1 inhibitors is likely to be limited (Figure S2).

Since our ^13^C-MRS studies showed a significant increase in glutamine flux to glutamate following mutant IDH1 inhibition, we considered using hyperpolarized glutamine to probe this pathway. Studies using hyperpolarized [5-^13^C] glutamine have been described in multiple tumor models 80-82. However, for our purposes, [5-^13^C] glutamine would likely be inadequate due to peak overlap for the C5 of glutamate and the C5 of 2-HG 83. Furthermore, work with hyperpolarized glutamine also presents a challenge due to the short T1 of the substrate (~8 s at 3T) and the rapid degradation of glutamine to glutamate 15, which would disrupt our ability to clearly quantify its metabolism to glutamate. A recent study described using hyperpolarized [1-^13^C] glutamine to probe 2-HG production in IDH1mut chondrosarcoma cells 83 and this approach could be considered. However, we chose to use hyperpolarized [1-^13^C] α-KG to probe our metabolic alterations because our group has previously shown its utility in IDH1mut tumors 35, 47 and it is being considered for clinical studies (Dan Vigneron, UCSF, personal communication).

Quantification of 2-HG production from hyperpolarized [1-^13^C] α-KG *in vivo* can be challenging due to the close resonance of the [1-^13^C] 2-HG and [5-^13^C] α-KG peaks. Nevertheless, studies in our lab using a 3T scanner, and thus a relatively long polarization for the ^13^C-labeled carbonyl, have demonstrated *in vivo* imaging of both [1-^13^C] 2-HG and [1-^13^C] glutamate production from hyperpolarized [1-^13^C] α-KG to distinguish IDH1mut tumors from IDH1wt in an orthotopic glioma rat model 35, 47. These studies used optimized sequences which maximized product signal and minimized substrate signal, allowing for detection of [1-^13^C] 2-HG via analysis of a bimodal temporal evolution of the overlapping peak 35. Further improvements could potentially be achieved by acquiring data at a single time point when the product 2-HG signal is expected to be maximal while the substrate hyperpolarized [5-^13^C]-α-KG would have decayed significantly.

Our study shows that hyperpolarized α-KG can be used to monitor IDH1mut inhibition by probing both the drop in 2-HG production and the increase in glutamate (Figure 4 and 5). The presence of these two hyperpolarized biomarkers, rather than only one, potentially allows for a more robust method of analysis. With one metabolite upregulated following treatment and the other downregulated, each biomarker confirms the validity of the other and reduces potential analytical bias. Such findings, combined with a ^1^H-MRS-detected increase in glutamate or Glx, would thus further strengthen the observation of a drop in 2-HG. These observations could also potentially address the challenges previously reported in AML where suppression of 2-HG alone was not sufficient to predict response to therapy 84, and potentially help in those cases when 2-HG levels are below detection, or in the small subset of IDH1mut glioma patients that lose the IDH1wt or IDH1mut allele resulting in loss of 2-HG production 85.

Further studies are required to confirm the application of our methods *in vivo* in animal models and most importantly in the clinical setting. Nonetheless, collectively, our MRS detectable metabolic changes could provide a set of complementary biomarkers to help assess the delivery and drug action of mutant IDH1 inhibitors, possibly predicting response to treatment.

## Conclusion

In summary, this study confirms the utility of ^1^H-MRS and identifies potential complementary hyperpolarized ^13^C-MRS biomarkers for assessing response to IDH1mut inhibition. Our observation that IDH1mut inhibition is associated with a unique MRS-detectable metabolic signature identifies a translational imaging approach that could be exploited for early non-invasive monitoring of response to IDH1mut inhibitor therapies in glioma patients.

## Supplementary Material

Supplementary figures and tables.

## Abbreviations

2-HG: 2-hydroxyglutarate
α-KG: alpha-ketoglutarate
AML: acute myeloid leukemia
AST: aspartate transaminase
BCAT1: branched chain aminotransferase 1
CT: computerized axial tomography
dDNP: dissolution dynamic nuclear polarization
GBM: glioblastoma
GDH: glutamate dehydrogenase
HIF: hypoxia-inducible factor
IDH: isocitrate dehydrogenase
IDH1mut: mutant isocitrate dehydrogenase 1
IDH1wt: wild-type isocitrate dehydrogenase 1
LGG: low-grade glioma
MRI: magnetic resonance imaging
MRS: magnetic resonance spectroscopy
NHA: normal human astrocyte
PDH: pyruvate dehydrogenase
PC: phosphocholine
WHO: world health organization
