# Supplementary material for: MR-detectable metabolic biomarkers of response to mutant IDH inhibition in low-grade glioma
Source: Theranostics. 2020 Jul 9;10(19):8757–70. doi: 10.7150/thno.47317 (PMC7392019; doi:10.7150/thno.47317)
Supplement: Supplementary file 1 — Supplementary figures and tables. [file thnov10p8757s1.pdf]

Supplementary Table 1. Quantification and significance of all ^{1}H-MRS-detectable metabolites in control and treated NHAIDH1mut cell extracts. Results are expressed as mean ± standard deviation.

	DMSO	AG-120	AG-120 v. DMSO p-value	1-Way ANOVA	AG-881	AG-881 v. DMSO p-value	1-Way ANOVA
Lactate	1.20 ± 0.37	1.25 ± 0.43	0.85	>0.99	1.27 ± 0.34	0.69	>0.99
Alanine	0.75 ± 0.12	0.68 ± 0.12	0.33	>0.99	0.85 ± 0.15	0.16	0.41
Acetate	0.49 ± 0.16	0.52 ± 0.17	0.71	>0.99	0.59 ± 0.17	0.23	0.68
2-HG	8.82 ± 1.06	0.21 ± 0.18	$2.54*10^{-9}$	$2.60*10^{-14}$	1.09 ± 0.33	$2.18 * 10^{-9}$	$1.50*10^{-14}$
Glutamate	3.90 ± 0.44	6.32 ± 0.67	$3.83*10^{-4}$	$3.49*10^{-4}$	7.21 ± 1.31	$1.07 * 10^{-4}$	$1.09*10^{-6}$
Glutamine	3.54 ± 0.57	3.25 ± 0.67	0.25	0.91	3.13 ± 0.46	0.12	0.29
Aspartate	1.33 ± 0.14	1.42 ± 0.17	0.31	>0.99	1.63 ± 0.37	0.07	0.09
Glutathione	1.31 ± 0.31	1.30 ± 0.24	0.97	>0.99	1.41 ± 0.14	0.36	>0.99
Choline	0.06 ± 0.02	0.05 ± 0.01	0.43	>0.99	0.04 ± 0.02	0.30	0.78
PC	0.63 ± 0.06	1.30 ± 0.21	$1.57*10^{-3}$	$8.25*10^{-4}$	1.46 ± 0.41	$6.52 * 10^{-4}$	$1.23*10^{-5}$
GPC	0.09 ± 0.04	0.10 ± 0.15	0.86	>0.99	0.05 ± 0.07	0.11	0.84
Myoinositol	2.47 ± 0.48	1.97 ± 0.44	0.08	0.19	2.17 ± 0.45	0.20	>0.99

Supplementary Table 2. Quantification and significance of all ^{1}H-MRS-detectable metabolites in control and treated U87IDH1mut cell extracts. Results are expressed as mean ± standard deviation.

	DMSO	AG-120	AG-120 v. DMSO p-value	1-Way ANOVA	AG-881	AG-881 v. DMSO p-value	1-Way ANOVA
Lactate	3.16 ± 0.91	3.83 ± 1.17	0.15	0.45	3.36 ± 1.34	0.67	>0.99
Alanine	0.88 ± 0.31	0.94 ± 0.12	0.53	>0.99	0.94 ± 0.49	0.72	>0.99
Acetate	0.74 ± 0.42	0.93 ± 0.41	0.27	0.70	0.84 ± 0.31	0.51	>0.99
2-HG	2.66 ± 0.55	0.29 ± 0.27	$2.37*10^{-13}$	$1.0*10^{-15}$	0.25 ± 0.29	$1.58*10^{-13}$	$1.0*10^{-15}$
Glutamate	4.04 ± 0.74	6.90 ± 0.90	$4.99*10^{-8}$	$1.59*10^{-11}$	7.26 ± 0.41	$2.41*10^{-13}$	$6.90*10^{-13}$
Glutamine	3.21 ± 1.21	2.96 ± 0.63	0.49	>0.99	4.10 ± 1.10	0.06	0.11
Aspartate	0.53 ± 0.15	0.47 ± 0.05	0.16	>0.99	0.58 ± 0.28	0.59	>0.99
Glutathione	2.99 ± 0.91	2.78 ± 0.86	0.57	>0.99	3.57 ± 0.45	0.07	0.20
Choline	0.11 ± 0.07	0.13 ± 0.06	0.40	>0.99	0.10 ± 0.04	0.60	>0.99
PC	1.60 ± 0.58	1.91 ± 0.23	$6.72*10^{-2}$	0.35	2.53 ± 0.54	$2.98*10^{-4}$	7.88*10-5
GPC	2.47 ± 1.1	2.56 ± 1.40	0.86	>0.99	2.58 ± 0.60	0.75	>0.99
Myoinositol	1.46 ± 1.07	0.90 ± 0.66	0.11	0.38	0.95 ± 0.86	0.18	0.48

Supplementary Table 3. Quantification and significance of all ^{1}H-MRS-detectable metabolites in control and treated NHAIDH1wt cell extracts. Results are expressed as mean ± standard deviation.

	DMSO	AG-120	AG-120 v. DMSO p-value	1-Way ANOVA	AG-881	AG-881 v. DMSO p-value	1-Way ANOVA
Lactate	1.54 ± 0.28	1.67 ± 0.46	0.68	>0.99	1.69 ± 0.16	0.45	>0.99
Alanine	0.47 ± 0.08	0.46 ± 0.06	0.93	>0.99	0.52 ± 0.06	0.43	>0.99
Acetate	0.24 ± 0.13	0.22 ± 0.14	0.87	>0.99	0.30 ± 0.11	0.61	>0.99
2-HG	0.17 ± 0.02	0.25 ± 0.05	0.06	0.10	0.23 ± 0.03	0.06	0.27
Glutamate	5.21 ± 0.72	5.09 ± 0.52	0.83	>0.99	5.70 ± 0.43	0.37	>0.99
Glutamine	3.49 ± 0.49	3.54 ± 0.28	0.87	>0.99	4.05 ± 0.32	0.17	0.34
Aspartate	0.90 ± 0.28	0.93 ± 0.41	0.94	>0.99	0.97 ± 0.34	0.80	>0.99
Glutathione	1.61 ± 0.45	1.43 ± 0.23	0.57	>0.99	1.44 ± 0.21	0.58	>0.99
Choline	0.09 ± 0.01	0.18 ± 0.14	0.33	0.81	0.13 ± 0.07	0.33	>0.99
PC	1.26 ± 0.35	1.25 ± 0.17	0.97	>0.99	1.42 ± 0.25	0.57	>0.99
GPC	0.11 ± 0.03	0.14 ± 0.06	0.47	>0.99	0.10 ± 0.05	0.83	>0.99
Myoinositol	1.98 ± 0.39	1.94 ± 0.30	0.91	>0.99	2.16 ± 0.33	0.56	>0.99

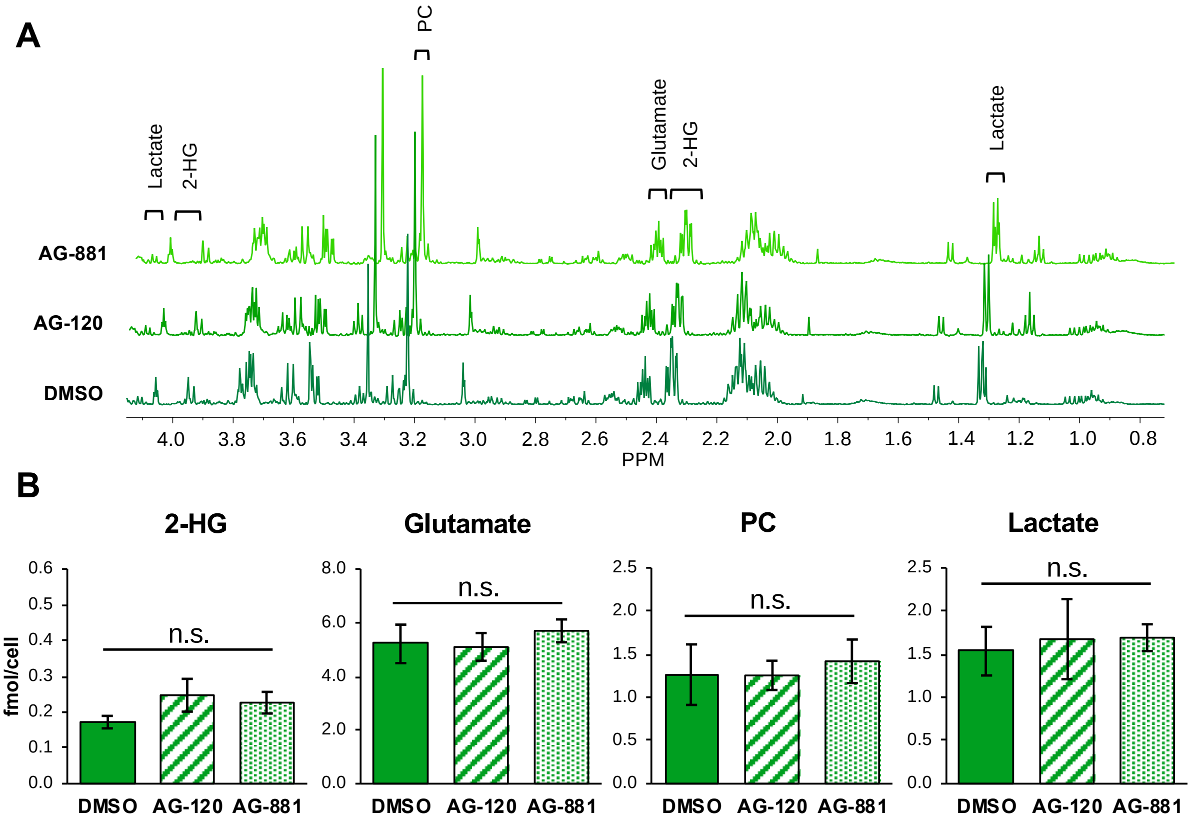

Supplementary Figure 1: **^{1}H-MRS spectra show that AG-120 and AG-881 do not alter steady-state metabolite levels in NHAIDH1wt cells. (A)** Representative ^{1}H-MRS spectra of NHAIDH1wt cells treated with DMSO, (bottom), AG-120 (center), and AG-881 (top). **(B)** Quantification of steady-state NHAIDH1wt metabolite concentrations. Results illustrate that AG-120 and AG-881 do not affect 2-HG, PC, glutamate, or lactate levels.

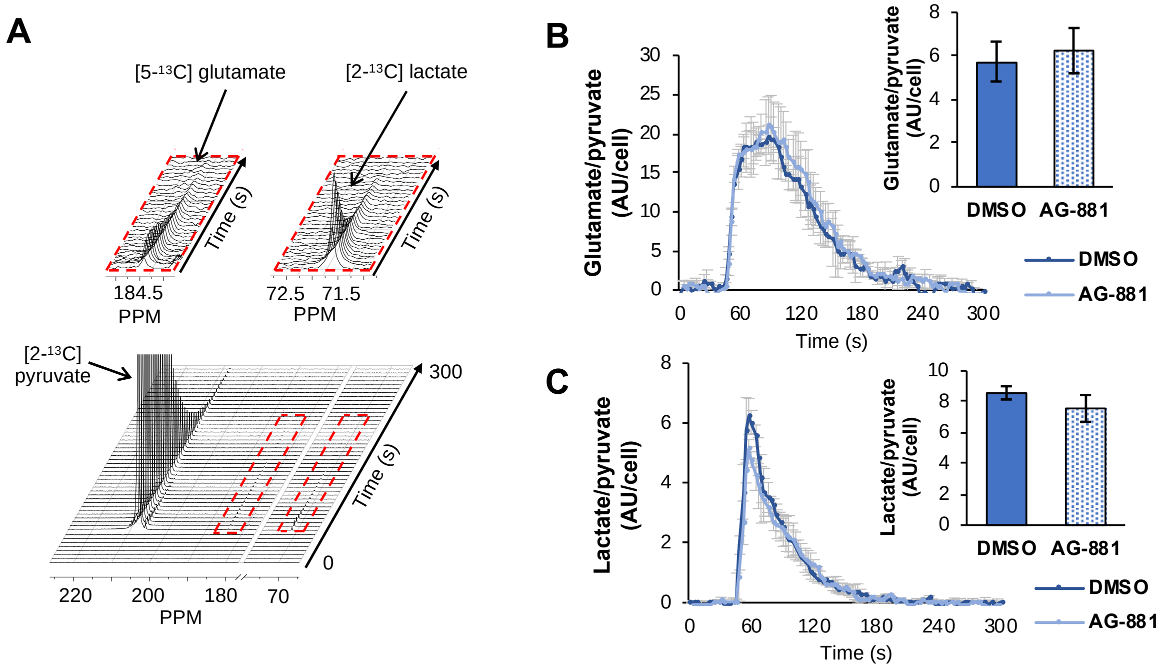

Supplementary Figure 2: **No change in fluxes from hyperpolarized [2-^{13}C] pyruvate to [5-^{13}C] glutamate or [2-^{13}C] lactate detected. (A)** Representative ^{13}C-MRS spectral array of [5-^{13}C] glutamate and [2-^{13}C] lactate production from hyperpolarized [2-^{13}C] pyruvate in live NHAIDHmut cells acquired at 1.5 Tesla (region of [5-^{13}C] glutamate and [2-^{13}C] lactate peaks expanded). **(B)** Quantification of ^{13}C-MRS spectra shows no change in [5-^{13}C] glutamate production or **(C)** in [2-^{13}C] lactate production following treatment with AG-881.

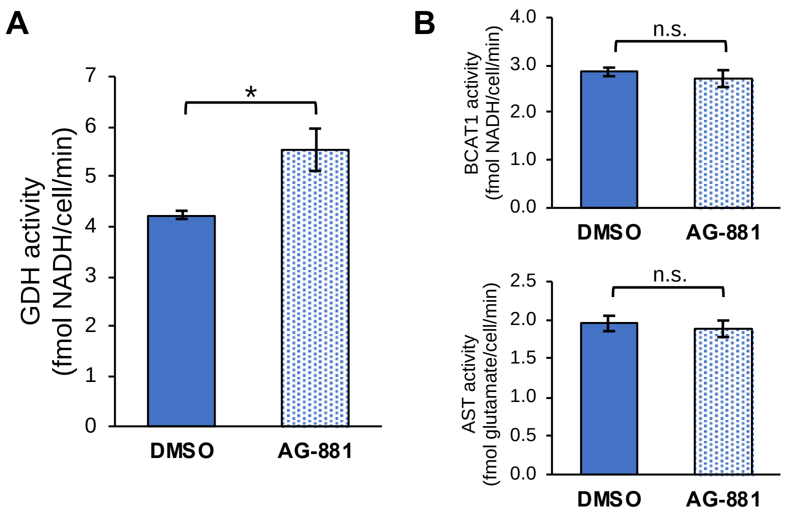

Supplementary Figure 3: **AG-881 treatment affects some of the enzymes that convert α-KG to glutamate. (A)** Spectrophotometric assay shows a significant increase in GDH enzyme activity in AG-881-treated cells. **(B)** Spectrophotometric assays show no significant change in BCAT1 or AST enzyme activity. AST: aspartate transaminase; BCAT1: branched chain aminotransferase 1; GDH: glutamate dehydrogenase.
